# The effects of topical cycloplegics in acute acquired comitant esotropia induced by excessive digital device usage

**DOI:** 10.1186/s12886-022-02590-w

**Published:** 2022-09-10

**Authors:** Rijo Hayashi, Shimmin Hayashi, Shigeki Machida

**Affiliations:** 1 Department of Ophthalmology, Saitama Medical Center, Dokkyo Medical University, 2-1-50 Minamikoshigaya, Koshigaya, Saitama 343-0845 Japan; 2 Lively Eye Clinic, Soka, Saitama Japan

**Keywords:** Acute acquired comitant esotropia, Excessive usage of digital devices, smartphone, Topical cycloplegics

## Abstract

**Background:**

Acute acquired comitant esotropia induced by excessive digital device usage, especially smartphones (SAACE), has been increasing over the past few years. One suggested mechanism is convergence spasm induced by excessive near work, with refraining from digital device use considered to be an effective method for decreasing the degree of esodeviation. However, if symptoms persist and are untreated over time, recovery becomes more difficult. The present study evaluated the effects of topical cycloplegics on persistent SAACE untreated for over 1 year.

**Methods:**

Patients with sustained SAACE that was untreated for over 1 year were evaluated. Digital device usage was discouraged and a topical cycloplegic, 0.4% tropicamide, was prescribed at bedtime. After obtaining informed consent, the 14 out of 23 enrolled patients who agreed to eye drop administration were defined as the study group, with the others serving as the controls. After a 3-month follow-up, patients who elected to undergo surgery were analyzed as the surgery group. Changes in esotropia angles, stereoacuity and diplopia complaints were evaluated after a 3-month follow-up.

**Results:**

Esotropia angles decreased and stereoacuity improved after a 3-month treatment in the study group (*P* < 0.01). Diplopia disappeared in 13 patients (92.9%, totally disappeared or disappeared when using glasses with built-in prisms). Among 11 patients with untreated esotropia ranging from 1–3 years, decreases in esotropia angles were correlated to untreated esotropia duration (near: *R* = -0.6; distance: *R* = 0.7; both *P* < 0.05). Esotropia angles in the control group exhibited a tendency to increase while stereoacuity tended to deteriorate after the 3-month follow-up. As diplopia did not disappear in any patients, 7 elected to undergo surgery and were enrolled as the surgery group. While esotropia angles decreased in the study group, they were lower than the surgery group (*P* < 0.01), but higher than the control group (*P* < 0.01). Stereoacuity was worse in the control versus the study and surgery groups (both *P* < 0.05).

**Conclusion:**

Results suggest short-acting topical cycloplegics are effective in SAACE patients with long untreated periods. Decreases in esotropia angles were negatively correlated to untreated esotropia duration, which suggests the necessity of early treatment.

## Background

Acute acquired comitant esotropia (AACE) is known as an acute onset of esotropia characterized by an equal angle of deviation in all fields of gaze [1, 2]. In addition to a known involvement with intracranial diseases, several other causes have been reported to potentially be connected to the development of AACE, with the majority of the causes associated with the acute accommodative type [3]. Increases in AACE have also been reported to be related to excess digital device usage, which includes smartphones, tablets and computers, with the usage of these devices concomitantly found to have also increased within the last decades [4–7]. During the Coronavirus disease 2019 (COVID-19) pandemic lockdowns, there was an increase in digital device usage by children due to both studying and playing. It was additionally reported that this AACE onset was correlated with this increased usage [8–10].

Furthermore, this type of AACE, smartphone associated AACE or SAACE, is thought to be induced by convergence spasm that follows excessive near work. It has been suggested that refraining from excessive near work can be effective in helping to decrease the degree of esodeviation. However, as there is limited remission after long untreated periods, it has been reported that surgical intervention can become necessary in some of these patients [4, 11].

Good results after surgical intervention have been reported for both AACE [12–15] and SAACE [4, 10]. However, there have yet to be any investigations into the effects of non-surgical treatments. Thus, our current case–control study was conducted to evaluate the effects of a non-surgical treatment, the use of topical cycloplegics, on SAACE patients with long untreated periods.

## Methods

All enrolled patients exhibited onset of esotropia after several hours of digital device usage, with the symptoms persisting for more than 1 year in the absence of any treatment. Patients with a history of ocular diseases, including strabismus and amblyopia, ocular surgery, and systemic diseases, including diabetes and neurologic diseases, were excluded. Patients with abnormal ocular movement were also excluded. All enrolled patients underwent brain and orbital computed tomography or magnetic resonance imaging to exclude intracranial and extraocular muscle abnormalities. Angles of esotropia were measured using the alternate prism cover test with near (33 cm) and distance fixation (6 m). Stereoacuity was measured using the Titmus Stereo test at 40 cm. Refractive errors were measured with 1% cyclopentolate hydrochloride.

**Fig. 1:**
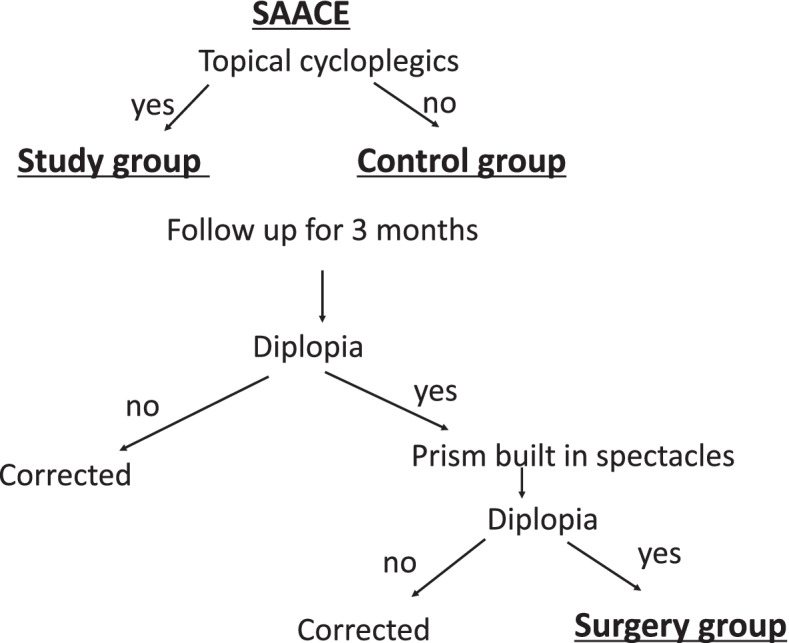
Protocol for dividing patients into groups. Enrolled patients were divided into the study and control groups according to whether they agreed to use eye drops. After 3 months of observation, surgery was performed on patients who still had sustained diplopia when using glasses with built-in prisms

After obtaining informed consent, all patients underwent baseline orthoptic examinations and then were divided into study and control groups based on whether they agreed to use eye drops. Figure 1 presents the protocol used to divide the patients into groups. Topical cycloplegics (0.4% tropicamide) were prescribed at bedtime every night in the study group. Both the study and control patient groups were advised to limit their digital device usage. At 3 months after the start of the study, angles of esotropia and stereoacuity were measured.

Diplopia improvement was also assessed at 3 months after the start of the study. For patients who were still complaining of diplopia, these subjects were prescribed glasses that contained built-in prisms, with 14 prism diopters the maximum. For patients still exhibiting sustained diplopia when using these glasses with the built-in prisms, surgery was performed and these patients were then enrolled in the surgery group.

In the study group, the correlation between the decreases in the angles of esotropia and the untreated periods (the period between onset and the beginning of treatment) was also investigated. In addition, the correlation between the decreases in the angles of esotropia and the amount of refraction errors were also measured. All procedures followed the tenets of the Declaration of Helsinki. Approval was granted by the Institutional Human Experimentation Committee of the Saitama Medical Center, Dokkyo Medical University (Approval number: 2070).

Comparisons between the angles of esotropia at baseline and those observed after the 3-month follow-up were analyzed using a paired *t*-test. As the results of the Titmus Stereo test are not evenly stepwise, the comparisons of the stereoacuity were analyzed used a paired *t*-test after logarithmic conversion. All correlations were analyzed using regression analyses. Significance was set at a *P*-value of 0.05.

## Results

Table 1 presents the characteristics on the 23 SAACE patients that were enrolled in the study and control groups. With the exception for the larger baseline angles of esotropia that were observed in the control group, there were no statistically significant differences between the study and control groups for the age of onset, age at the start of treatment, untreated period, baseline stereoacuity and refraction errors.

**Table 1:** Characteristics of patients enrolled

	**Study group** (*n* = 14)	**Control group** (*n* = 9)
	mean ± SD (range)	mean ± SD (range)
Age of onset(y/o)	20.0 ± 7.3 (13 ~ 38)	21.3 ± 13.6 (3 ~ 48)
Age on treatment(y/o)	22.6 ± 7.4 (14 ~ 39)	25.0 ± 14.3 (4 ~ 50)
Untreated period (duration, yrs)	2.6 ± 2.3 (1 ~ 10)	2.1 ± 1.1 (1 ~ 4)
Angle of estropia (⊿)^a^		
at near	23.6 ± 12.0 (8 ~ 45)	33.3 ± 15.4 (20 ~ 70)
at distance	25.6 ± 14.4 (8 ~ 50)	34.4 ± 10.7 (25 ~ 60)
Stereoacuity^b^	5.49 ± 1.55 (3000 ~ 40 s)	5.39 ± 1.69 (> 3000 ~ 40 s)
Refraction errors (D)^c^	-4.76 ± 2.58 (0 ~ -8.25)	-4.11 ± 4.23 (+ 3.00 ~ -8.50)

^a^Measured with Alternate Prism Cover Test (APCT）

^b^Measured with Titmus Stereo Test, logarithmic conversion

^c^Measure with 1% cyclopentolate hydrochloride

**Fig. 2:**
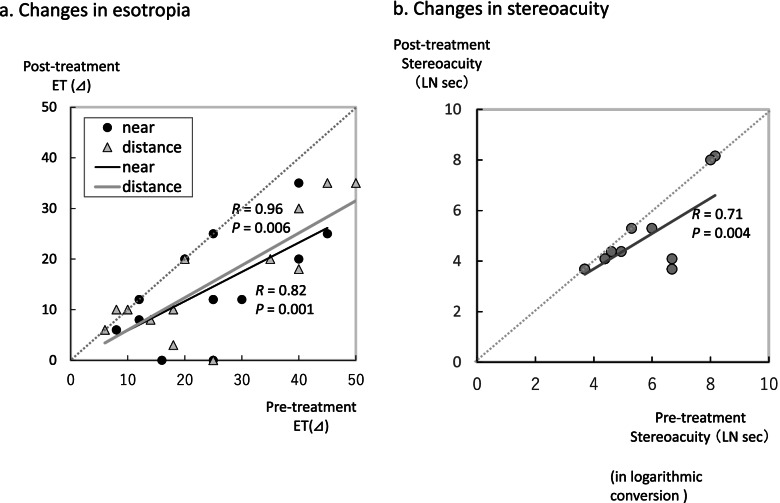
Changes in the study group. **a** Changes in the angles of esotropia. In the study group, there were significant associations between the pre-and post-treatment angles of esotropia. There were no increases in the angles of esotropia in the study group. **b** Changes in the stereoacuity in the study group. In the study group, there were significant associations between the pre-and post-treatment stereoacuity. There was no deterioration of the stereoacuity in the study group

**Fig. 3:**
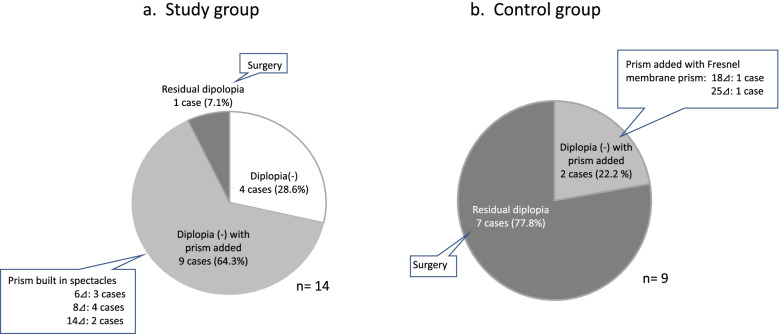
Improvement of diplopia. **a** In the study group, diplopia disappeared in 4 patients (28.6%). Diplopia was corrected using glasses with built-in prisms in 9 patients (64.3%, 3 with 6⊿, 4 with 8⊿, and 2 with 14⊿). **b** In the control group, all 9 patients in the control group had sustained diplopia that could not be corrected when using glasses with built-in prisms. Of these, 2 patients who declined surgery underwent corrections using a Fresnel membrane prism. The other 7 patients who did undergo surgery were included in the surgery group

**Fig. 4:**
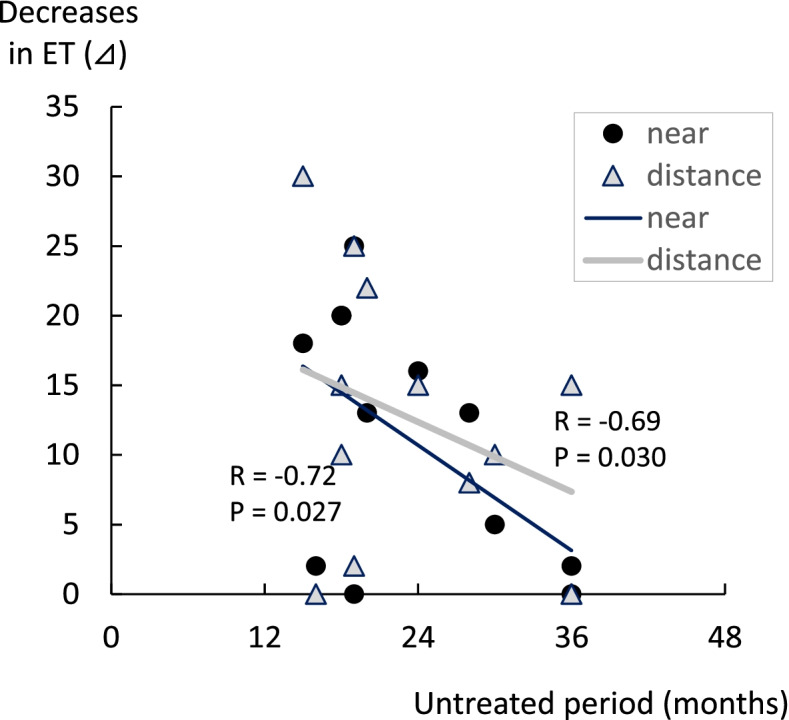
Correlation between untreated periods and decreases in the angles of deviation in the study group. Only patients with untreated periods less than 3 years were included in the analyses of the correlation. Decreases in the angles of esotropia were negatively correlated to the untreated period, with the longer the untreated period, the less the observed decreases in the angles of esotropia

**Fig. 5:**
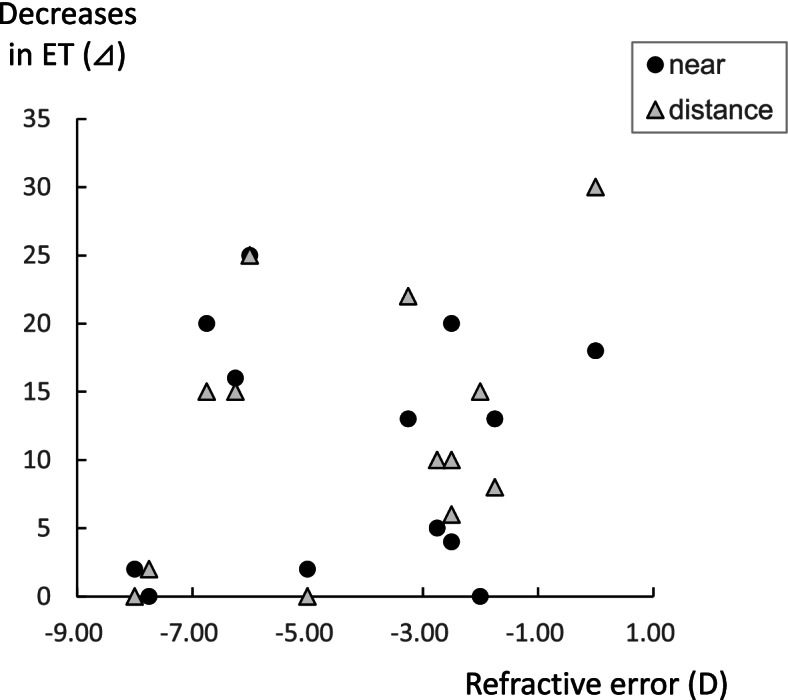
Correlation between refraction errors and decreases in the angles of deviation in the study group. There was no correlation between the refraction errors and decreases in the angles of deviation in the study group

There was a significant decrease in the angles of esotropia, both near and distance fixation in the study group after the 3-month treatment (Table 2, Fig. 2a). For the stereoacuity, there was a significant improvement observed after the treatment (Fig. 2b). Among the 14 patients in the study group, diplopia disappeared in 4 patients after the 3-month treatment (Fig. 3). In 9 patients, the diplopia disappeared when using the glasses with the built-in prism, while there was 1 patient who still exhibited sustained diplopia, thereby requiring subsequent surgery. As only 2 patients were examined in the study group, it is likely that more patients will need to be evaluated in order to investigate the correlation between the decreases in the angles of esotropia and the untreated periods that were longer than 3 years. In our current analyses of the correlation, only patients with untreated periods less than 3 years were included. Decreases in the angles of esotropia were found to be negatively correlated to the untreated periods (Fig. 4), with smaller decreases in the angles of esotropia found for the longer untreated periods. The decrease in the angles of esotropia was not correlated to the amount of refraction errors (Fig. 5).

**Table 2:** Changes after 3-month observation

**Study group**	**Pre-treatment**	**Post-treatment**	***P*** **value of paired** ***t*-test**
Angles of esotropia (⊿)			
at near	23.6 ± 12.0	13.8 ± 10.1	0.0012
at distance	25.6 ± 14.4	15.8 ± 11.7	0.0019
Stereoacuity	5.49 ± 1.55	4.73 ± 1.52	0.0296
**Control group**	**Baseline**	**3 months later**	***P* value of paired *t*-test**
Angles of esotropia (⊿)			
at near	33.3 ± 15.4	37.8 ± 19.1	n.s
at distance	34.4 ± 10.7	35.9 ± 11.8	n.s
Stereoacuity	5.39 ± 1.69	5.80 ± 1.86	n.s

**Fig. 6:**
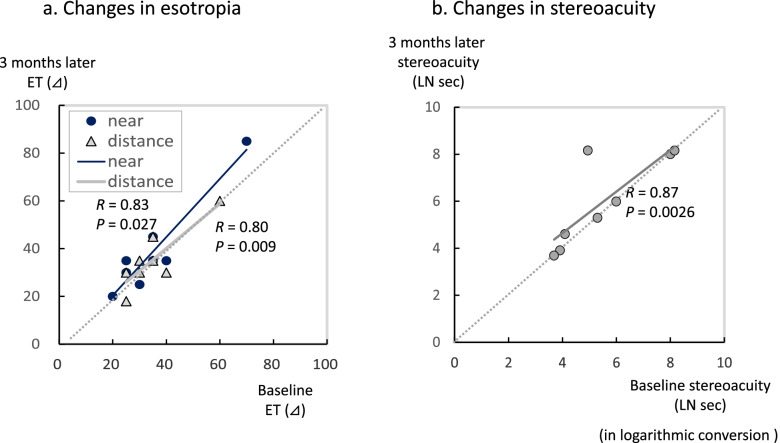
Changes in the control group. **a** Changes in the angles of esotropia. There were significant associations between the pre-and post-treatment angles of esotropia. There was a tendency for an increase in the angles of esotropia at near fixation after a 3-month observation period. **b** Changes in the stereoacuity in the control group. There were significant associations between the pre-and post-treatment stereoacuity. There was a tendency for deterioration in the stereoacuity after a 3-month observation period

After 3 months of observation, there was a tendency for an increase in the angles of esotropia in the control group (Table 2, Fig. 6a). Also, there was a tendency for deterioration in the stereoacuity (Fig. 6b). All 9 patients in the control group exhibited sustained diplopia that could not be corrected when using the glasses with the built-in prisms (Fig. 3b). In the 2 patients who declined surgery, they underwent correction using a Fresnel membrane prism. In the other 7 patients in the control group, they agreed to undergo surgery and thus, were included in the surgery group.

**Fig. 7:**
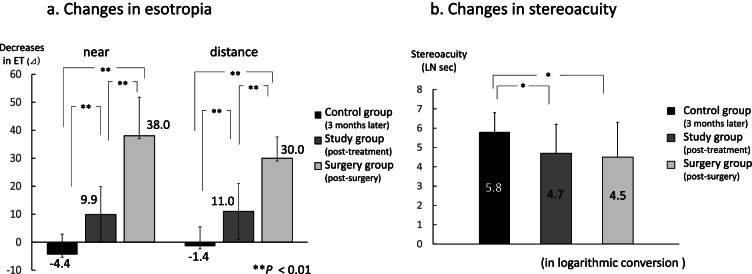
Comparisons among the study, control and surgery groups. **a** Comparisons of decreases in the angles of deviation. Compared to the findings observed in the surgery group, while the decreases in the angles of esotropia in the study group were lower, they were higher than that observed in the control group for both the near and distance fixation. **b** Comparisons of stereoacuity. Compared to the findings observed in the control group, the stereoacuity in the study group was better, with a level similar to that observed in the surgery group

As compared to the surgery group, the decreases in the angles of esotropia in the study group were lower. However, these were higher compared to that observed in the control group (Fig. 7a). Furthermore, the stereoacuity in the study group was better as compared to the control group, with levels that were the same as that found in the surgery group (Fig. 7b).

## Discussion

After 3 months of topical cycloplegics, stereoacuity improved following the esotropia decreases among the SAACE patients. However, the administration was found to be less effective in patients with longer untreated periods.

Three major types of AACE have been defined [1]: (1) Swan type: esotropia due to the disruption of fusion following monocular occlusion or vision loss in young children; (2) Burian-Franceschetti: esotropia associated with physical or psychological stress in young patients; (3) Bielschowsky type: esotropia in young uncorrected myopia with excessive near work. Excessive near work with excessive accommodation followed by convergence spasm has been suggested to be the mechanism of this esotropia [16]. Since the convergence spasm in this condition cannot be relaxed at distance fixation, there is development of esotropia with diplopia. The imbalance between the convergence and divergence forces also leads to the development of increases in the tonus of the medial rectus, which leads to esotropia. Patients enrolled in this study exhibited the onset of AACE after several hours of digital device usage. Only 1 out of the 23 patients had emmetropia. The other 22 patients had myopia, with 8 of these patients exhibiting high myopia over -6.0D. The characteristics found for these patients were similar to those of Bielschowsky esotropia, which exhibits equal deviation at near and distance fixation, myopia and adolescent onset after excessive near work.

Changes in the tonus of accommodation and binocular vergence have been reported after near work [17], especially after hours of work with digital devices [18, 19]. As compared to that found for ordinary hard copy workers, digital devices workers exhibit a higher ratio of abnormal accommodation and convergence [20]. Over the last few decades, the usage of handheld devices, including smartphones and tablets, has increased, especially during lockdowns due to the COVID-19 pandemic. Several ocular symptoms, including dry eye and binocular disabilities have also been reported after excessive usage of handheld devices [21, 22]. When using a smartphone, the working distances are shorter than that found for typical near working distances [23], with these distances becoming even shorter when there is over 60 min of usage [24]. Furthermore, some subjects will view text on their smartphones using a smaller font size [23]. Deterioration of fusional vergence has been reported after 30-min smartphone usage [25]. When smartphone or tablet usage was evaluated in AACE patients, nearer working distances were found as compared to that observed in age-matched controls [11]. Thus, closer working distances and smaller font sizes may increase the demands on accommodation and convergence, thereby resulting in a spasm of the near reflex that leads to dynamic activation of the medial rectus. It has been suggested that spasm of the near reflex could be one of the causes in patients presenting with AACE [26].

Anatomical anomalies could also potentially contribute to the onset of SAACE. It has been reported that there are shorter distances between the limbus and the insertion of the medial recti in AACE [13]. Hypertrophic medial recti were also observed in some of the patients in our surgery group. In addition, the changes of the resting states of accommodation and vergence in near work have been reported to depend on the oculomotor resting tonus [17]. Additional investigations into the possibilities of anomalies in the extraocular muscles, such as the distance between the limbus and insertion, will need to be undertaken in the future.

In our current study, in addition to imposing limitations on digital device usage, a prescription of 0.4% tropicamide at bedtime was found to improve the angles of deviation and diplopia among patients with untreated SAACE that had persisted for over 1 year. As we have described earlier, excessive accommodation and convergence could potentially contribute to the SAACE onset. Cycloplegic eye drops, which include cyclopentolate and atropine, have been reported to be effective on releasing spasm of the near reflex, especially the accommodation component [27]. Moreover, the prescription of atropine has also been reported to resolve Bielschowsky esotropia [16]. It has additionally been reported that 2 cases of AACE with accommodative spasm responded to 1% cyclopentolate eye drops [28] and 1% atropine sulfate eye ointment [29]. Cycloplegics inhibit spasm of the ciliary muscle and near reflex, and thus, can further improve esotropia. Tropicamide has been reported to have the similar cycloplegic effects as cyclopentolate [30–32], although tropicamide has a shorter duration as compared to that observed for cyclopentolate and atropine sulfate. The onset of SAACE is thought to be due to the continuity of the convergence spasm after excessive near work. A 0.4% tropicamide prescription at bedtime appears to inhibit the accommodative spasm during the night. Although these effects do not persist during the following day, once the accommodative spasm stops, the continuity of accommodative spasm is interrupted. Accompanying convergence spasms also stopped at night when the continuity was interrupted. As a result, the diplopia disappeared in conjunction with decreases in the esotropia. Moreover, a prescription of 0.4% tropicamide at bedtime will not induce mydriasis or cycloplegia during the daytime. Thus, this could reduce patient distress and increase treatment compliance.

There were several limitations in the present study. First, the current results suggest both imposing limitations on the digital device usage and the administration of topical cycloplegics could be beneficial in these types of patients. Further evaluation of the individual effects of 0.4% tropicamide will need to be undertaken. However, studies done to examine the amount of time spent using digital devices can only be performed based on self-reported data. In this study, there were equal proportions of patients in both the study and control groups who reported the same daily average time of digital device usage, which was over 80%, even after being advised to limit their usage. Thus, duration of smartphone usage affected the study group in the same way as that observed in the control group and therefore, it is not likely that this influenced the results of the present study. Furthermore, our results also suggest that topical cycloplegics can help resolve SAACE. Although decreases in the angles of deviation were less in patients using topical cycloplegics, the angle of esotropia decreased to near 50%. Accompanying this decease, there was an improvement in the stereoacuity, with patients achieving the same level as that observed for patients undergoing surgery. Furthermore, using glasses with built-in prisms was able to correct diplopia in most patients (13 out of 14 patients) in conjunction with cycloplegic eye drops. Thus, overall, the findings of our present study indicate the beneficial effects of topical cycloplegics in treating SAACE, especially in patients who are not able or do not agree to undergo any operations. The small sample size is another limitation of the present study. Although SAACE has recently garnered more attention, SAACE is still considered to be an unusual presentation of esotropia. Several case reports [5, 8] and case series [4, 9–11] have been published, with the largest series evaluating 15 patients [10]. Although our present study enrolled 23 patients with SAACE, which is a much larger number than that reported in previous studies, further evaluation of additional patients will be necessary in order to better investigate the characteristics and treatment issues associated with SAACE.

In our current study, 26% of the enrolled patients were over 25 years of age, which was higher than that reported in the other studies. Recently, excessive near work has become a more widely investigated issue, as this is now considered to be a major problem in not only teenagers but also in adults. According to our current results, there was no correlation between the age of onset and the decreases in the angles of esotropia after the administration of local short acting cycloplegics. Furthermore, tropicamide eye drops appear to be effective in patients up to the 4^th^ decade. Further studies that focus on patients of different age groups will need to be undertaken.

## Conclusions

In addition to limiting digital device usage, topical cycloplegics appear to be a promising treatment for acute esotropia induced by excessive usage of digital devices. However, these treatments are less effective in patients with longer untreated periods. Therefore, early diagnosis and treatment are important in these types of patients.

## Data Availability

The datasets generated and analyzed during the current study are not publicly available due to the privacy policy included in the informed consent but are available from the corresponding author on reasonable request.

## Abbreviations

AACE: Acute Acquired Comitant Esotropia
COVID-19: Coronavirus disease 2019
SAACE: AACE due to excess usage of smartphone
